# Genotoxic and cytotoxic potential of methacrylate-based orthodontic adhesives

**DOI:** 10.1007/s00784-020-03569-x

**Published:** 2020-09-24

**Authors:** Andreas Taubmann, Ines Willershausen, Christian Walter, Sarah Al-Maawi, Bernd Kaina, Lina Gölz

**Affiliations:** 1 Department of Operative Dentistry, Johannes Gutenberg University Hospital Mainz, Mainz, Germany; 2 Department of Orthodontics and Orofacial Orthopedics, Friedrich-Alexander University Erlangen-Nürnberg, Erlangen, Germany; 3 MEDI+ Mainz, Private Practice, Mainz, Germany; 4 Frankfurt Orofacial Regenerative Medicine (FORM) Lab, Department for Oral, Cranio-Maxillofacial and Facial Plastic Surgery, Medical Center of the Goethe University Frankfurt, Frankfurt/Main, Germany; 5 Institute of Toxicology, University Medical Center Mainz, Mainz, Germany

**Keywords:** γH2AX assay, Orthodontic adhesives, Methacrylates, DPIHP, Biocompatibility

## Abstract

**Objectives:**

The biocompatibility of methacrylate-based adhesives is a topic that is intensively discussed in dentistry. Since only limited evidence concerning the cyto- and genotoxicity of orthodontic adhesives is available, the aim of this study was to measure the genotoxic potential of seven orthodontic methacrylate-based adhesives.

**Materials and methods:**

The XTT assay was utilized to determine the cytotoxicity of Assure Plus, Assure Bonding Resin, ExciTE F, OptiBond Solo Plus, Scotchbond Universal Adhesive, Transbond MIP, and Transbond XT after an incubation period of 24 h on human gingival fibroblasts. We also performed the γH2AX assay to explore the genotoxic potential of the adhesives within cytotoxic dose ranges after an incubation period of 6 h.

**Results:**

The XTT assay showed a concentration-dependent reduction in cell viability. The decrease in cellular viability was in the same dose range most significant for Assure Plus, rendering it the adhesive material with the highest cytotoxicity. Employing the γH2AX assay, a concentration-dependent increase in H2AX phosphorylation was detected, indicating induction of DNA damage.

**Conclusions:**

For most products, a linear correlation between the material concentration and γH2AX foci was observed. The most severe effect on γH2AX focus induction was found for Transbond MIP, which was the only adhesive in the test group containing the co-initiator diphenyliodonium hexafluorophosphate (DPIHP).

**Clinical relevance:**

The data indicate that orthodontic adhesives, notably Transbond MIP, bear a genotoxic potential. Since the study was performed with in vitro cultivated cells, a direct translation of the findings to in vivo exposure conditions should be considered with great diligence.

## Introduction

The inception of adhesive techniques to orthodontics in the late 1960s has resulted in a focus on resin materials [1, 2]. Adhesive materials are currently well-established in orthodontic practice and are routinely used when applying fixed buccal/lingual bracket systems, attachments or retainers [3–5]. When dental materials are applied within the oral cavity, their biological safety is crucial to the clinical success of the respective treatment [6]. During the course of orthodontic bonding in particular, when the supernatant adhesive is homogenized by means of air pressure, monomers will come into close contact with the marginal gingiva, the vestibular mucosa or even become diluted in the aqueous oral cavity. It has been previously questioned whether the application of adhesives prior to bracket placement, escape of adhesive materials from the bracket base, incomplete removal from the tooth surface, and insufficient light curing pose a direct hazard to the oral environment [7]. As a consequence, the capacity of methacrylate-based dentin adhesives to exert adverse local or even systemic effects is of increased interest [8, 9]. With regard to the toxicity of different orthodontic adhesives, chemically cured liquid-paste materials have been confirmed to be more cytotoxic than light-cured materials [10]. Previous findings suggest the use of single cured systems since they appear to be superior to dual-cured systems in terms of a higher degree of conversion, less leaching and therefore lower cytotoxicity [11].

Bonding agents have further been investigated with regard to their effect on different cell lines. They have been reported to disrupt the cellular redox state of pulp cells in monolayer cultures and induce apoptosis and cell-cycle arrest of MDPC-23, undifferentiated pulp cells (OD-21), and macrophages [12–15]. With regard to the type of monomer, BisGMA has been shown to be highly toxic in close proximity to gingival fibroblasts, and TEGDMA induces apoptosis in cultured hGFs in a dose- and time-dependent manner [11, 16, 17]. HEMA was further shown to provoke accumulation of cells in the S-phase caused by DNA damage, and both HEMA and BisGMA induce oxidative DNA damage [18, 19].

The literature describes multiple methacrylate DNA-damaging mechanisms. A direct DNA effect, in which methacrylate carbon-carbon double bonds interact with the nucleophilic centers of the DNA, has been discussed as a mechanism behind [20]. Another possible mechanism is the induction of reactive oxygen species (ROS), which leads to oxidation products of DNA bases and DNA strand breaks. DNA breakage occurs during the process of DNA repair, notably through base excision repair (BER) removing oxidized DNA lesions such as 8-oxo-guanine. Overlapping single-strand breaks that arise from oxidative damage and overlapping BER patches can further cause DNA double-strand breaks (DSB) due to replication fork collapse [21]. In vitro experiments showed that antioxidants, such as ascorbic acid and N-acetylcysteine, reduce the level of DSBs following treatment with methacrylates, suggesting that DNA damage relies on the induction of ROS and the formation of epoxides rather than on the direct interaction of methacrylates (e.g., BisGMA, HEMA, TEGDMA, and UDMA) with the DNA [16, 20, 22, 23]. Due to increased knowledge regarding DNA repair and damage responses, γH2AX-dependent approaches have evolved as a reliable tool for the detection of DNA damage in dental material sciences [22, 24]. Nikolova et al. pointed out that prior to testing materials with regard to their genotoxicity, their respective cytotoxicity should be assessed in order to establish the proper dose range [25]. Based on recent evidence [16, 23, 26], we decided to evaluate the genotoxic potential of seven commercially available light-cured, single-bottle orthodontic adhesives (Assure Plus, Assure Bonding Resin, ExciTE F, OptiBond Solo Plus, Scotchbond Universal Adhesive, Transbond MIP, and Transbond XT). Although polymerized dentin adhesives are known to exhibit a lower cytotoxicity than their unpolymerized counterparts, we consciously chose to test the adhesives in their uncured form to adhere to the in vivo conditions within the oral cavity.

In addition to the XTT and Live/Dead assay to determine the cytotoxicity, we employed the γH2AX foci assay for detecting possible genotoxic effects induced by the materials. Since γH2AX can be considered a reliable bio-indicator for pretoxic DNA damage such as DSB and blocked replication forks [25], we investigated seven different orthodontic adhesive materials with respect to their ability to provoke γH2AX formation in human gingival cells.

## Materials and methods

### Cell culture

Human gingival fibroblasts (hGFs), derived from fragments of marginal gingiva harvested during the osteotomy of third molars, were isolated and cultured. Isolated hGFs were characterized by their spindle-shaped morphology and by expression of typical marker genes like fibronectin, vimentin, and Tuj-1 [27, 28]. Healthy volunteers (two males and two females, aged 15–18 years) were informed prior to treatment and agreed on donating explant material for experimental purposes. The study was conducted in accordance with the second Helsinki Declaration and was approved by the Ethics Committee of the University of Mainz. Isolated gingival tissue, free of epithelium and alveolar bone, was incubated in Dulbecco’s Modified Eagle’s Medium (DMEM) (Sigma, Taufkirchen, Germany) supplemented with 10% fetal calf serum (Sigma, Taufkirchen, Germany), 1% penicillin/streptomycin, and 1% fungizone (Sigma, Taufkirchen, Germany) at 37 °C and 5% CO_2_. After 3 weeks of incubation, primary colonies had formed. The cells were reseeded and cultivated in DMEM supplemented with 10% fetal calf serum (Sigma, Taufkirchen, Germany) and 1% penicillin/streptomycin at 37 °C and 5% CO_2_. For passaging and seeding of the cells, trypsin/EDTA solution was used (Sigma, Taufkirchen, Germany). Subsequent experiments were performed with cells from passage numbers 4–7.

### XTT proliferation assay

The cytotoxic potential of seven methacrylate-based adhesives was determined using the Cell Proliferation Kit II (XTT) (Roche, Mannheim, Germany). The working principle is to detect the conversion of tetrazolium salts into photometrical detectable formazan dye by the reducing potential of the outer surface of intact cell membranes, and therefore the presence of a viable cell population [29, 30]. The day before the experiment, hGFs were seeded into 96-well plates (Greiner, Frickenhausen, Germany) (10.000 cells per well). On the day of the experiment, liquid adhesives (Assure Plus, Pelz & Partner; Assure Bonding Resin, Pelz & Partner; ExciTE F, Ivoclar Vivadent; OptiBond Solo Plus, Kerr; Scotchbond Universal Adhesive, 3M Espe; Transbond MIP, 3M Unitek; Transbond XT, 3 M Unitek) were diluted 1:10 (v/v) in DMEM, avoiding direct exposure to light. The resulting predilution was applied to adherently growing hGFs in the 96-well plates (Greiner, Frickenhausen, Germany) at dilution factors of 1:3, 1:10, 1:30, 1:100, and 1:300 (v/v) and incubated for 20 h. Subsequently, the wells were washed once with phosphate-buffered saline (PBS) (Sigma, Taufkirchen, Germany), and fresh cell culture medium was added. The activated XTT reagent, as specified by the manufacturer, was applied to the wells and incubated at 37 °C and 5% CO_2_ for 4 h. Specific photometric absorbance of the formazan dye was measured at 450 nm and 670 nm as a reference wavelength, using the Synergy HT microplate reader (Biotek, Winooski, USA). As a control, cells were treated with cell culture medium. For the specimen Assure Sealant, Excite F, Optibond Solo, Scotchbond Universal, Transbond MIP, and Transbond XT, six experiments and therefore six individual values have been combined (*n* = 6). For the specimen Assure Plus, nine experiments and therefore nine individual values have been combined (*n* = 9). The treated specimens were normalized to the untreated control cells. To estimate the half maximum effect (EC50) of the tested material in terms of cytotoxicity/cell viability, a four-parameter logistic model was computed [31].

### Live/Dead assay

To validate the outcome of the EC50 estimation, e.g., determination of a specimen dose with a half maximum effect in terms of cell viability, the Live/Dead cell viability assay (Molecular Probes, Carlsbad, CA) was performed. For visual identification of viable cells with intact cell membranes, the green fluorescent dye calcein was utilized. In contrast, cells without a competent cell membrane, which were considered damaged or dead, provided an insufficient diffusion barrier for the ethidium homodimer. Therefore, this fluorescent dye can intercalate into the cell nucleus and emit a red fluorescent signal. For the Live/Dead assay, hGFs were seeded in 24-well plates (40.000 cells per well) on the day before the experiment. On the day of the experiment, the previously determined effective concentration (EC50), a higher (× 3) and a lower concentration (× 1/3) of the specimen, was applied to the cells. After incubation for 20 h, the cells were washed one time with PBS (Sigma, Taufkirchen, Germany) and stained as specified by the manufacturer. Stained cells were viewed using an inverted microscope Axiovert 40 CFL (Carl Zeiss, Jena, Germany) at wavelengths of 450/520 nm at a × 100 magnification, and images were captured using an Axio Cam MRC and Axiovision software (Carl Zeiss, Jena, Germany).

### γH2AX assay

Human gingival fibroblasts were seeded (75.000 per well) on coverslips (Knittel, Braunschweig, Germany) in 6-well plates (Greiner, Frickenhausen, Germany) 2 days before the experiment. On the day of the experiment, stock solutions of the adhesives were prepared by mixing the adhesive liquids at a 1:10 ratio with DMEM (10% FCS) (*v*/v). The medium in the culture vessels was removed, and dilutions of the stock solution at a ratio of 1:20, 1:60, and 1:180 were added to the cultured cells. As a positive control, 1 mM, 0.33 mM, or 0.1 mM methyl methanesulfonate (MMS) (Sigma, Taufkirchen, Germany) were added directly to the cell culture medium. After 1 h, the coverslips were washed once with PBS and incubated for another 6 h in fresh cell culture medium. As a control, fresh culture medium was added to the cells. After a 6-h treatment/recovery time, the cells were fixed using a 15-min treatment with 4% paraformaldehyde (Merck, Darmstadt, Germany) in PBS (Sigma, Taufkirchen, Germany).

Cells were washed once with PBS and permeabilized with ice-cold methanol (Merck, Darmstadt) at − 20 °C for 10 min. The specimens were rehydrated by three washing steps with PBS for 5 min and incubated with a blocking reagent (3% BSA, 0.25% Triton X-100 in PBS) for 1 h. Primary antibody (mouse anti-phospho-H2AX Ser 139, #05–636, Millipore) was diluted 1:1000 in PBS 0.25% Triton X-100 and incubated overnight at 4 °C. After three washing steps with PBS for 5 min, the secondary antibody diluted to 1:500 in PBS 0.25% Triton X-100 was incubated for 1 h at room temperature. After three washing steps with PBS for 5 min, the cells were incubated with 0.4 μg/ml DAPI (Roche, Mannheim) in ddH_2_O for another 5 min. Single cover glasses were mounted with 2-μl antifade medium (Vectashield) and nail polish on microscope slides. Microscopic images of the cell nuclei were acquired using the Axio Imager M1 (Carl Zeiss, Jena, Germany) and Metafer 4 software (Metasystems, Altlussheim, Germany). Fluorescence signals of DAPI and Alexa Fluor 488 were detected in 5 planes with a 0.5-μm focus plane distance at a magnification of × 1000. Using Metafer 4 software, the single focus planes were computed into z-projections for automated focus analysis. To score the γH2AX focus levels per cell, we used ImageJ (Fiji) as an automated focus counting tool, a method previously described by Nikolova et al. [25]. Three individual experiments were conducted, analyzing 80 cells per experiment and applied concentration, therefore obtaining two hundred forty individual values (*n* = 240). The individual values were summarized as means and the standard deviations were calculated.

### Statistical analysis

For analysis of the XTT assay, the quantitative study data were examined by analysis of variance (ANOVA) with correction by Tukey post hoc assessment to compare the groups using SPSS Statistics software 23.0 (SPSS Inc., Chicago, USA). Analysis of data derived from the yH2AX assay was performed by group comparisons using the Kruskal-Wallis test with SPSS Statistics software 23.0 (SPSS Inc., Chicago, USA). Differences were considered significant if the *p* values were less than 0.05 (*p* < 0.05).

## Results

### Effect of methacrylate-based adhesives on cell viability

**Fig. 1:**
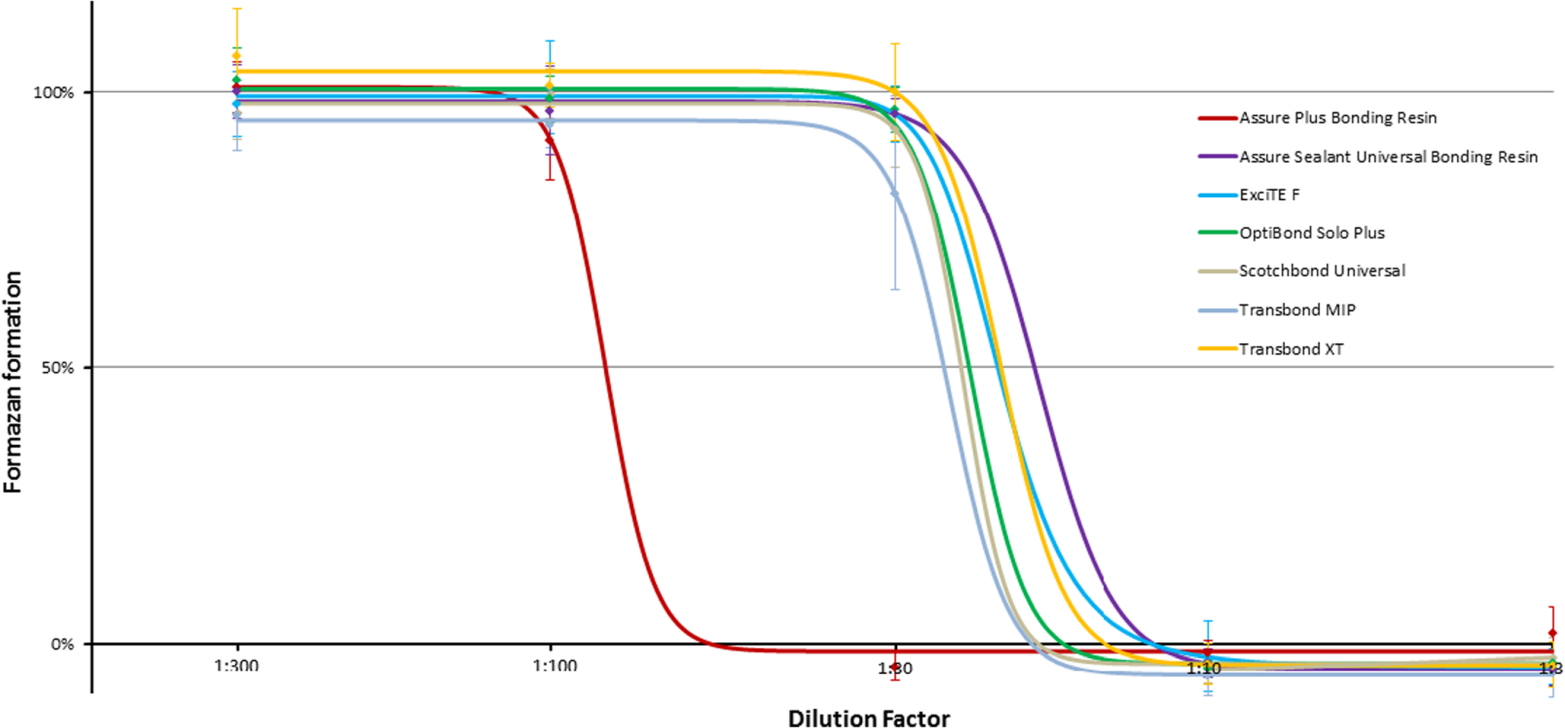
Dose response curve for cell viability after treatment of hGfs with different dilutions of adhesives. The sigmoidal curve is the result of the computation via a four-point logistic function to estimate the effective concentration (EC50). The EC50 represents the dilution factor corresponding to the half maximum effect in terms of formazan formation compared with the control (Table 1). The mean levels of formazan formation in cells treated with the respective dilution of different adhesives after 24 h of exposure is shown as diamonds. The presented values have been normalized to untreated control cells (*n* ≥ 6). Error bars represent the standard deviation of the mean (SDM)

With an increasing concentration of the adhesive, all the investigated specimens showed a steady decrease in cell viability after 24 h. At the highest and lowest adhesive concentrations, the cell viability reached a plateau. For Assure Bonding Resin, ExciTE F, OptiBond Solo Plus, Scotchbond Universal Adhesive, Transbond MIP, and Transbond XT, the most pronounced decrease in cell viability was observed as dilutions of 1:10 and 1:30 were added to the cells. For Assure Plus Bonding Resin, a major reduction in cell viability was observed with a dilution range from 1:100 to 1:30. None of the tested products showed a significant amount of formazan formation at a dilution of lower than 1:10, indicating complete cell death at this high concentration level. To differentiate the adhesives based on their cytotoxicity, their relative toxicity was determined. Therefore, data from the XTT assay were employed to compute a sigmoidal dose-effect curve with the assistance of a four-point logistic model curve fitting algorithm [31] (Fig. 1). As an indicator of the validity of the fitted curve in relation to the previous measurements, the root mean square error (RMSE) was calculated. The RMSE was < 0.02 for all the fitted curves.

**Table 1:** Composition of the adhesive materials

Product	Supplier	Composition	%	CAS
Assure Plus All Surface	Pelz & Partner	Bisphenol A glycidyl dimethacrylate	20–50	1565-94-2
Bonding Resin		Ethanol	30–50	64-17-5
LOT 163091		10-Methacryloyldodeylphosphate	5–25	85590-00-7
		2-Hydroxyethyl methacrylate	5–25	868-77-9
Assure Sealant Universal	Pelz & Partner	Acetone	50–75	67-64-1
Bonding Resin		2-Hydroxyethyl methacrylate	10–30	868-77-9
LOT 156787		Bisphenol A glycidyl dimethacrylate	10–30	1565-94-2
		Proprietary monomer	10–30	
ExciTE F dental adhesive	Ivoclar Vivadent	Bisphenol A glycidyl dimethacrylate	25–50	1565-94-2
LOT V08590		Ethanol	10–< 25	64-17-5
		2-Hydroxyethyl methacrylate	10–< 25	868-77-9
		Propenoic Acid	10–< 25	223681-84-3
		Diurethane dimethacrylate	3–< 10	72869-86-4
		Potassium fluoride	0.3–< 1	7789-23-3
OptiBond Solo Plus	Kerr	Ethanol	10–30	64-17-5
LOT 5365766		2-Hydroxyethyl methacrylate	10–30	868-77-9
		2-Hydroxy-1,3-propanediyl bismethacrylate	1–5	1830-78-0
		Alkali fluorosilicates (Na)	0.1–1	16,893-85-9
Scotchbond Universal	3 M Espe	2-Hydroxyethyl methacrylate	15–25	868-77-9
Adhesive		Bisphenol A glycidyl dimethacrylate	15–25	1565-94-2
LOT 631026		Ethanol	10–15	64-17-5
		Ingredients without classification	10–15	
		Silane-treated silica	5–15	122334-95-6
		Decamethylendimethacrylate	5–15	6701-13-9
		2-Proprionic acid, 2-methyl-reaction products with		
		1,10-Decanediol and phosphorus oxide (P2O5)	1–10	1207736-18-2
		Copolymer of acrylic and itaconic acid	1–5	25,948-33-8
		Campherquinone	< 2	10373-78-1
		N,N-Dimethylbenzocaine	< 2	10287-53-3
		(Dimethylamino) ethylmethacrylate	< 2	2867-47-2
		Butanone	< 0.5	78-93-3
Transbond MIP	3 M Unitek	Ethanol	30–40	64-17-5
LOT N775853		Bisphenol A glycidyl dimethacrylate	15–25	1565-94-2
		2-Hydroxyethyl methacrylate	10–20	868-77-9
		2-Hydroxy-1,3-propanediyl bismethacrylate	5–15	1830-78-0
		Copolymer of acrylic and itaconic acid	5–15	25,948-33-8
		Diurethane dimethacrylate	1–10	72,869-86-4
		Water	1–10	7732-18-5
		Diphenyliodonium hexafluorophosphate	< 1	58109-40-3
		N,N-Dimethylbenzocaine	< 0.5	10287-53-3
		Triphenylantimony	< 0.5	603-36-1
Transbond XT	3 M Unitek	Bisphenol A glycidyl dimethacrylate	45–55	1565-94-2
Light Cure Adhesive Primer		Triethylene Glycol Dimethacrylate	45–55	109-16-0
LOT N763457		Triphenylantimony	< 1	603-36-1
		4-(Dimethylamino)-benzene ethanol	< 0.5	50438-75-0
		Campherquinone	< 0.3	10373-78-1
		Hydroquinone	< 0.03	123-31-9

Names of substances specified in variable manners by the manufacturers but with corresponding CAS numbers have been adjusted

The half maximum effect of a substance in terms of cell viability is referred to as the concentration at which formazan formation is half maximum compared with the controls (EC50). Considering the fitted curve, the EC50 value corresponded to the point immediately between the upper and lower plateau and the point with the highest slope, respectively. The EC50 was lowest for the adhesive Assure Sealant Universal Bonding Resin (Dilution factor 17.9), and hence this product was considered to be the least toxic, immediately followed by Transbond XT, Scotchbond Universal Adhesive, ExciTE F, Optibond Solo Plus, and Transbond MIP (Table 2). Notably, concentrations of Assure Plus 4.37-fold lower than those of Assure Sealant were equally effective.

**Table 2:** Effective concentration and relative toxicity (means of the EC50 values) of the tested dental adhesives

Product	Effective concentration EC50 ± SDM	Relative toxicity
Assure Sealant Universal Bonding Resin ^*^	17.9 ± 4.3	1
Transbond XT ^*^	20.2 ± 4.5	1.13
Scotchbond Universal Adhesive ^*^	20.6 ± 3.9	1.15
ExciTE F ^*^	22.1 ± 2.6	1.23
OptiBond Solo Plus ^*^	22.7 ± 1.6	1.27
Transbond MIP ^*^	23.8 ± 3.3	1.33
Assure Plus Bonding Resin	78.2 ± 9.9	4.37

^*^Significantly different than Assure Plus Bonding Resin (*p* ≤ 0.05)

For the specimen Assure Sealant, Excite F, Optibond Solo, Scotchbond Universal, Transbond MIP, Transbond XT six experiments were performed (*n* = 6) and for Assure Plus nine experiments were carried out (*n* = 9)

**Fig. 2:**
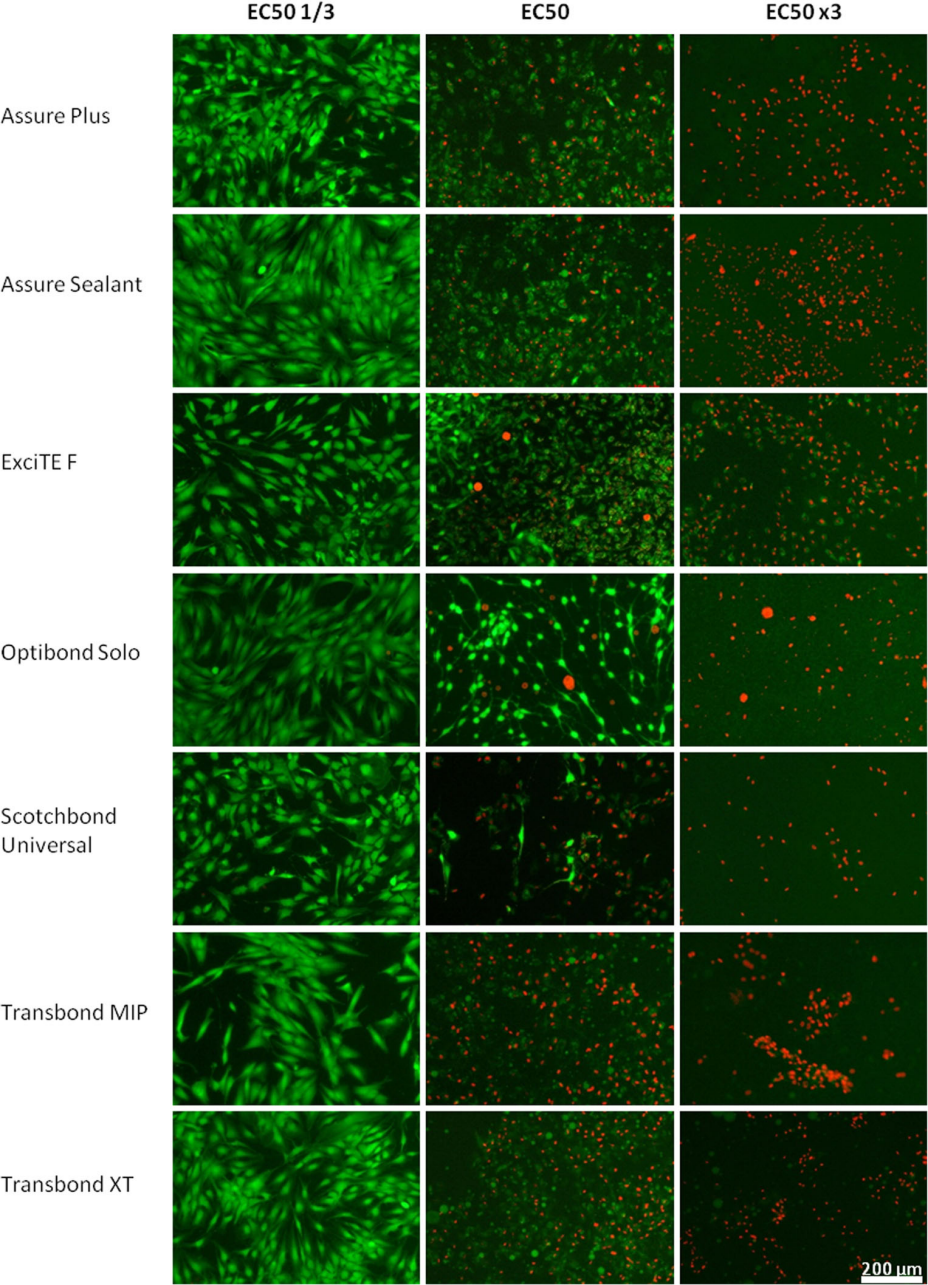
Human gingival fibroblasts 24 h after incubation with dilutions of dental adhesives. Staining was performed in accordance with the Live/Dead assay. Calcein stains living cells with intracellular esterase activity fluorescent green due to enzymatic conversion. Ethidium homodimer intercalates into DNA of dead or dying cells, causing a red fluorescent signal, while being excluded from living cells. Live/Dead cells and morphology were evaluated to validate the estimated effective concentration (EC50) of the material (*n* = 3)

To validate the estimated EC50 values, microscopic investigation of the cell monolayer utilizing the Live/Dead assay was performed (Fig. 2). Dilutions of the adhesives were used that corresponded to the calculated EC50. In addition, a lower (× 1/3) and a higher (× 3) concentration were utilized to represent the upper and lower plateau of the relevant sigmoidal curve. For all tested products, the EC50 × 1/3 concentration did not alter the cell viability and resembled a healthy proliferating cell culture without any ethidium homodimer staining after 24 h of exposure.

A good correlation between the applied conditions of cell monolayer culture and microscopic evaluation, regarding the distribution of dead/dying cells when applying the EC50, was found for Assure Plus, Assure Sealant, ExciTE F, Transbond MIP, and Transbond XT. A portion of cells showed red nuclear staining, representing the dead fraction of the cell population, but also showed dim green cytoplasmic staining. The other part of the population exhibited dim green staining, without the red nuclear staining, most likely representing cells with residual enzymatic activity and intact cellular membrane, therefore counted as alive. Significantly, both types demonstrated an altered, condensed cell morphology compared with the specimen with EC50 × 1/3 treatment.

The morphology of the cells treated with Optibond Solo and Scotchbond Universal at EC50 dilution differed from the other respective samples. Despite displaying viable and dead cells, the cell morphology was less homogeneous, partly rounded up and stretched, with some exhibiting bright green cytoplasmic staining.

Cells that had been treated with the EC50 × 3 dilution of the corresponding adhesive were exclusively stained red and counted as dead. Only the ExciTE F specimen exhibited dim cytoplasmic in addition to nuclear staining. In the case of the EC50 × 3 dilution of Transbond XT, all cell nuclei were stained with the ethidium homodimer. Notably, large round blobs with dim green fluoresce remained in the specimen. Likely as a consequence of the fluorescent signals in the blue channel (not depicted), the blobs were considered to be residual adhesive coating the cell culture vessel, which was also visible in the EC50 sample.

### Elucidating the genotoxic potential of the dental adhesives via the γH2AX assay

Taking into account that the previously tested adhesives showed cytotoxic effects depending on a rather broad range of adhesive dilutions (1:17.9 to 1:78.2), we attempted to compare the potential genotoxic effects of the substances. Of note, the clinician would likely apply considerably similar amounts of material during treatment, irrespective of the utilized product. Therefore, we attempted to test for potential genotoxic effects with similar dilutions of the different products as they might be present in the aqueous environment of the oral cavity. A dilution of the prepared stock solution by the factor of 1:20 was set as a starting concentration with subsequent dilutions by a factor of 3, testing 1:60 and 1:180 dilutions, respectively.

**Fig. 3:**
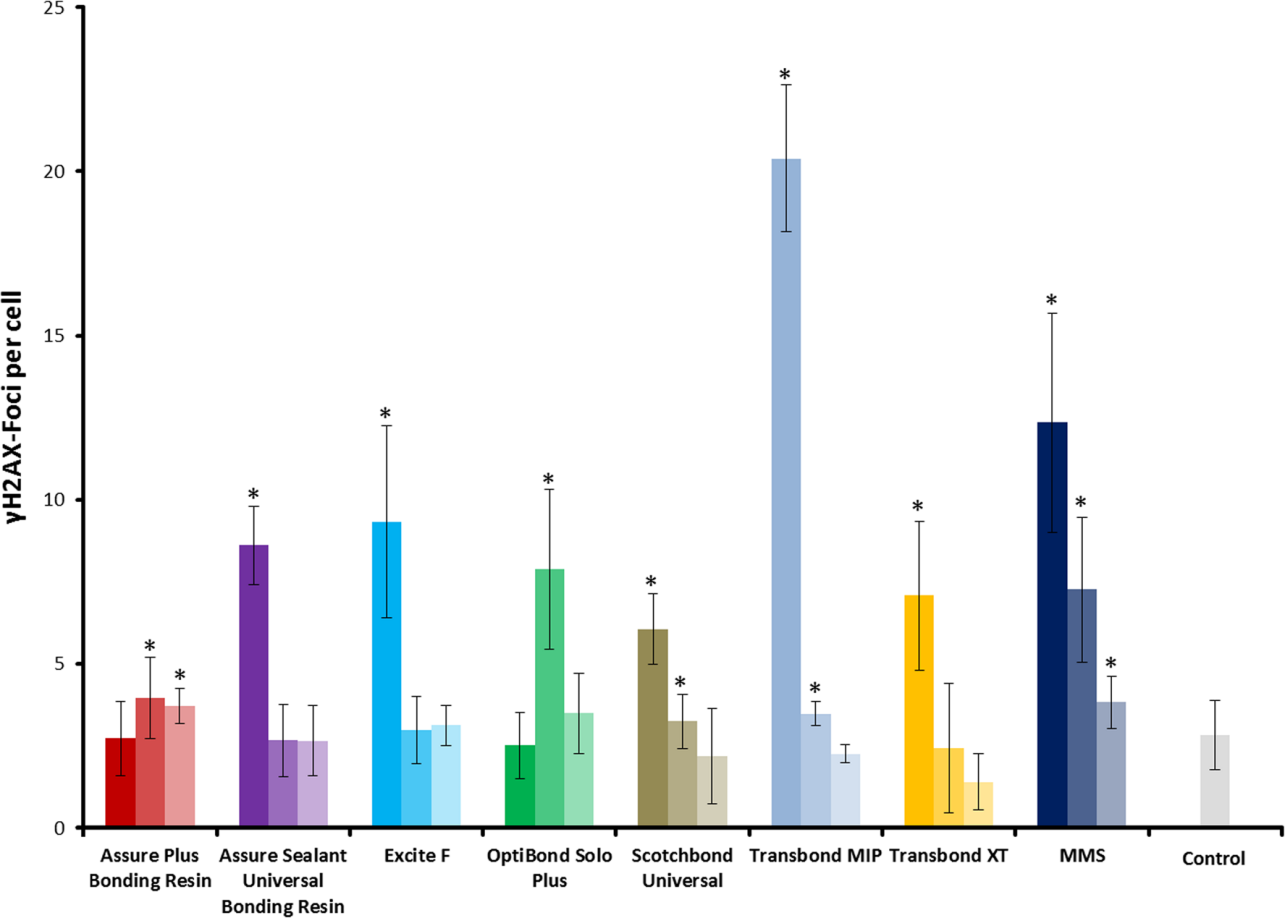
Induction of γH2AX foci in human gingival fibroblasts after 6 h of incubation with different methacrylate-based adhesives. The color shading refers to the different concentrations used, corresponding to 1:20, 1:60, and 1:180 for the adhesives and 1 mM, 0.3 mM, and 0.1 mM of methyl methanesulfonate (MMS) as a positive control, respectively. The highest concentration is depicted as saturated color, and lower concentrations are indicated by lighter shades of the corresponding color. The columns represent the mean values with the standard deviation of the mean between experiments (SDM). Data from three independent experiments each analyzing 80 nuclei are shown (*n* = 240). Asterisks highlight significant differences compared with the control (*p* ≤ 0.05)

**Fig. 4:**
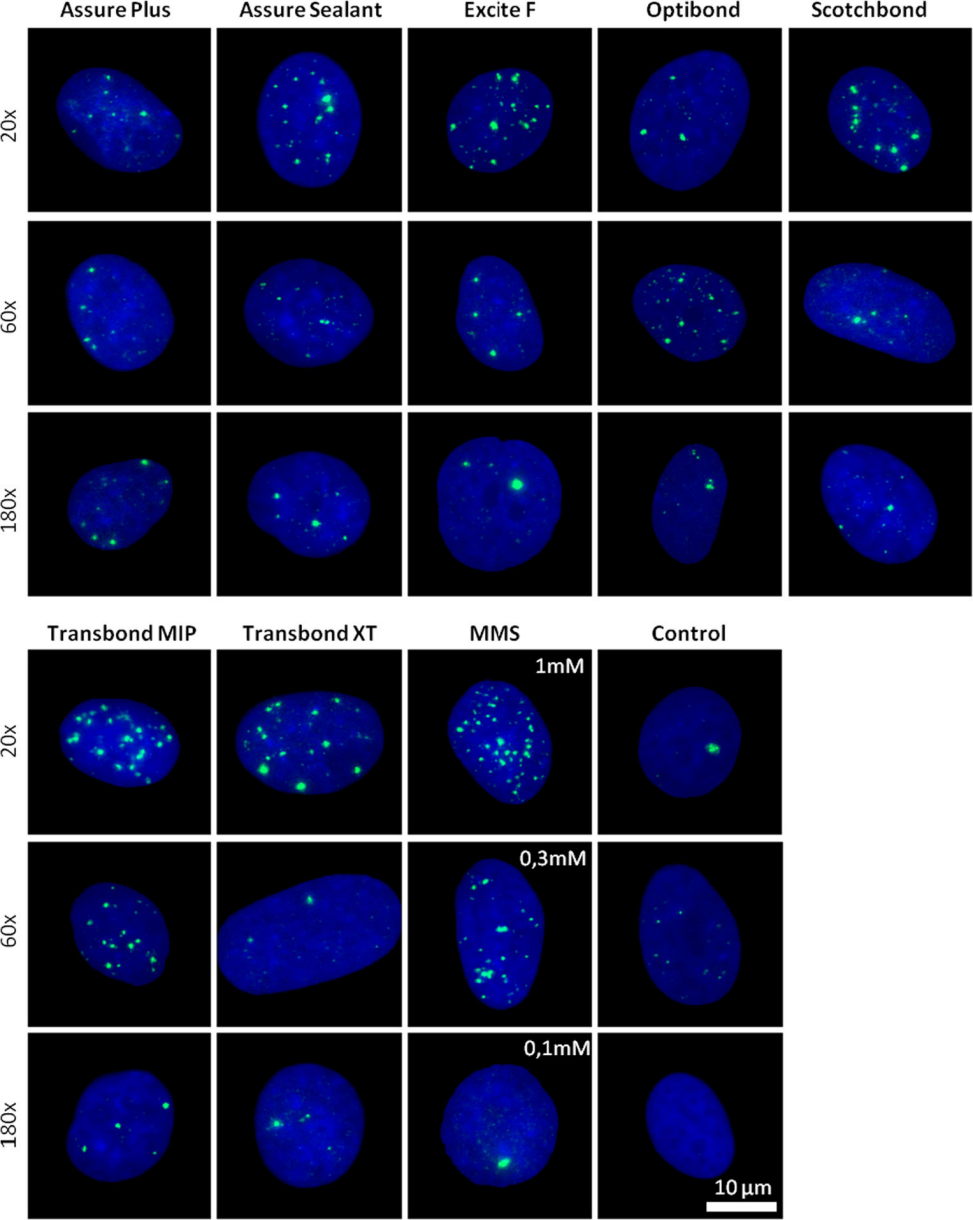
Representative images of γH2AX focus staining in fixed human gingival fibroblasts. The cells were treated with the indicated dilution of the adhesive supernatant for 6 h. As positive control, cells were treated with the indicated concentrations of the genotoxin methyl methanesulfonate (MMS) for 1 h followed by 6 h recovery. γH2AX foci are depicted in green. Nuclear counterstaining was performed with DAPI and is depicted in blue. Control, non-treated cells

The γH2AX foci numbers in the samples incubated with the highest adhesive concentrations (1:20) were for Assure Plus 2.7 ± 1.1 (mean ± SDM), for Assure Sealant 8.6 ± 1.2, for Excite F 9.3 ± 2.9, for Optibond Solo Plus 2.5 ± 1, for Scotchbond Universal 6 ± 1, for Transbond MIP 20.3 ± 2.2, and for Transbond XT 7 ± 2.4. A positive control was 1-mM MMS, for which 12 ± 3.3 foci/cell were determined (see (Fig. 3 and Fig. 4)). Untreated control cells displayed on average 2.8 ± 1 foci per cell. For most of the samples, the largest amount of induced γH2AX foci was measured when the highest concentration of the monomers was used for treatment.

Only for products Assure Plus and Optibond, the highest foci numbers were not detected in the sample with the highest concentration. For Assure Plus, a significant increase in the foci level compared with the control could only be detected in the samples with the higher dilution rates, with 3.9 ± 1.2 at 1:60 and 3.7 ± 0.5 at 1:180. For Optibond Solo Plus, the highest number of foci could be detected in the 1:60 sample (10.5 ± 2.4). The highest level of focus induction was observed in the samples treated with the highest concentration of Transbond MIP (20.3 ± 2.2), indicating Transbond MIP has the highest genotoxic potential.

## Discussion

The DNA double strand break is considered to be the most dangerous lesion impacting a cell’s genomic integrity. A well-orchestrated system of DNA damage sensors, signal transmitters, and executors either inhibit cell cycle progression and provide sufficient time for DNA damage repair or induce apoptosis in order to prevent transformation into a cancer cell. The key factors for DNA damage recognition are the kinases ATM and ATR, which phosphorylate and therefore activate subsequent proteins and induce the DNA damage response, with p53 playing a pivotal role [32]. Whether the DNA damage response machinery is activated by double-strand breaks or indirectly by stalled replication forks, one of the very early reactions to these insults is the phosphorylation of the histone subvariant H2AX into γH2AX conducted by the kinases ATM and ATR. Both kinases facilitate the regulation of p53, the so-called guardian of the genome, either by direct or indirect interaction via mediating kinases.

Most of the findings regarding the discrimination of genotoxic or non-genotoxic compounds inducing cytotoxic effects can be applied to isolated substances. Therefore, discriminating a mixture of several known genotoxicants and/or non-genotoxicans with regard to their cytotoxicity is complex. Both cytotoxicities due to preceding DNA damage and direct cytotoxic events are involved, while other damaging mechanisms leading to apoptosis, necroptosis, or necrosis have to be taken into account as well.

The present study was conducted to investigate the cytotoxicity and the genotoxic potential of seven commercially available orthodontic methacrylate-based, light-cured adhesives using the XTT, the Live/Dead assay, and γH2AX assay, respectively. Since the isolated adhesive compounds, predominantly methacrylate monomers, were shown to impact the genomic integrity, we addressed the question of whether methacrylate-based products of varying composition exert a risk regarding the induction of DNA damage. Several oral cell culture models attested that monomers such as BisGMA, HEMA, TEGDMA, and UDMA showed elevated cytotoxicity and induced oxidative stress, i.e., ROS formation [17, 26, 33–36].

The small monomer HEMA has hydrophilic properties and is therefore frequently added to adhesives to ensure good wetting of dental surfaces and further enhances handling when combined with hydrophobic or highly viscous monomers like BisGMA [37].

HEMA induces oxidative stress accompanied with reduced cell viability due to depletion of the cytoprotective metabolite GSH. The reversal of the particular phenotype by antioxidants acting as ROS scavengers like N-acetylcysteine, ascorbate, or melatonin is underlining that ROS formation is key to a decreasing cell survival [19, 22, 38]. The dose-dependent increase of the cleavage of pro-caspase 3, yielding the critical apoptosis effector caspase 3, adds further evidence to the hypothesis of an apoptosis-dependent decrease in cell viability [38]. The disruption of the mitochondrial membrane potential in cell cultures incubated with HEMA suggests that resin monomer-induced programmed cell death can be mediated through the intrinsic mitochondrial pathway of apoptosis [38]. Additionally, p53-dependent pathways of apoptosis have been shown for HEMA. Earlier reports indicated the formation of DNA double-strand breaks in HEMA exposed cells [17, 18], leading to genotoxic or mutagenic events most likely based on oxidative DNA damage [39].

In general, the cytotoxic potential of a monomer is dependent on its specification and the respective dose employed. As DNA double-strand breaks can lead to apoptosis, the amount of detected γH2AX foci directly correlates with a reduction of viability of the treated cells. Monomers such as BisGMA, UDMA, TEGDMA, and HEMA show differences in terms of cytotoxicity and genotoxicity, with the most severe effects found in BisGMA, followed by UDMA, TEGDMA, and HEMA [17]. It was assumed before that these differences can be attributed to different liposolubility, with BisGMA sharing the highest liposolubility and enabling a more effective cell penetration [17]. In addition to a ROS- dependent mechanism of DNA damage induction, epoxy methacrylates have been detected in methacrylate-treated cells as well [40, 41]. The assumption that methacrylate monomers are metabolized into epoxy compounds and exert a direct DNA damage induction has been recently substantiated [17, 23]. Throughout the present study, we used primary gingival fibroblasts, which represent a potential target for the monomers mentioned above. First, we assessed human fibroblasts incubated with liquid adhesives and observed a reduction in cell viability to various extent. Morphological aspects typical of apoptosis such as pseudopod retraction, rounding up of the cells and a decreased cellular volume (pyknosis) were observed at concentrations determined to be cytotoxic in the XTT assay. These results are in accordance with previous reports on dental adhesive methacrylates [16, 26]. The resulting DNA damage can be detected by the comet assay, micronucleus assay, chromosomal aberration, sister-chromatid exchange, and the γH2AX assay, all of which are used to analyze different genotoxic end points [42]. Here, we utilized the γH2AX foci assay and assessed the genotoxicity of the materials by comparing the adhesive materials at the same dose level as to its ability to induce DNA damage. It is worth mentioning that the XTT or Live/Dead assay and the γH2AX assay require different incubation times. The XTT assay investigates cells after an incubation period of 24 h, while the γH2AX assay examines cells after 6 h of exposure to the diluted adhesive. The early γH2AX detection allows an assessment even though the dose applied could be cytotoxic. This feature is of particular importance since in late apoptotic stages, e.g., nuclear fragments or micronuclei; the γH2AX signal is not always linked to a DSB induced by the agent in question [16]. Thus, apoptotic events may have an impact on the proper detection of γH2AX foci. Furthermore, strong pan nuclear γH2AX staining was shown to result from massive DNA cleavage due to caspase-activated nuclease [43, 44]. To avoid mistaking those events for one another, only intact nuclei were evaluated, and cells exhibiting pan nuclear γH2AX staining were excluded from the analysis. For AP, we observed no increase in γH2AX foci formation at high-dose levels. This could be due to cell cycle perturbations. Although vital cells were still present after treatment, the cells could be arrested in specific cell cycle phases, preventing from the formation of DSBs, and therefore γH2AX foci. This might lead to cell cycle-specific killing of cells in vulnerable cell cycle phases, such as the S-phase. Evidence for this assumption has been previously presented in a report on cell cycle effects of dental adhesives on human gingival fibroblasts [19]. The adhesives induced an increase in the G1/G0 and G2/M populations, which was accompanied by a depletion of the S-phase population. When examining the composition of Assure Plus, attention is drawn to 10-methacryloyldodeylphosphate (10-MDP), a compound that is solely present in this adhesive. The 10-MDP represents a functional monomer with a self-etch function, which is able to form a stable calcium salt, and thereby adhere to enamel surfaces. It has been reported that impurities in different 10-MDP formulas influence the biocompatibility of the material [45]. However, in our setting, whether the compound itself or potential impurities were responsible for its cytotoxic potential remained elusive. The pronounced cytotoxicity of OptiBond Solo likely diminished the amount of evaluable viable or actively cycling cells, and therefore interfered with the proper detection of γH2AX at the highest concentration. Considering the Live/Dead assay, the growth pattern of the monolayer as well as the cell morphology suggested an elevated cytotoxicity of the adhesive associated with rounding up of the cells. The remaining cell membrane integrity might provide a hint concerning why the level of cytotoxicity was not portrayed to the same extent by the XTT assay, despite ongoing apoptotic processes. Likewise, the most prominent increase in γH2AX focus induction as observed at lower adhesive concentrations.

For Assure Sealant, Excite F, Scotchbond Universal, and Transbond XT, which show comparable cytotoxicity in the XTT and Live/Dead assay, a linear relationship between the adhesive concentration and γH2AX focus induction was apparent. However, Transbond MIP, the cytotoxicity of which is comparable with the aforementioned adhesives, was distinguished by a significant increase in yH2AX numbers.

While the photoinitiator camphorquinone (CQ) was listed in two samples, the co-initiator diphenyliodonium hexafluorophosphate (DPIHP), an iodonium salt releasing the active compound diphenyliodonium (DPI), was only listed for Transbond MIP. For DPI, the intracellular mode of action in terms of ROS induction seems to be paradox, since both the stimulation and the inhibition of ROS have been reported in literature [46–48]. The potential of DPI as an inhibitor of the NADPH oxidase with subsequent diminishment of ROS production on the one hand and the disturbance of the cellular redox balance including depletion of the GSH pool on the other hand suggests a concentration-dependent mode of action, with a shift toward induction of oxidative stress at higher concentrations. Another DNA damaging mechanism has been proposed for DPI as well, via the acceptance of an electron at redox centers like the aforementioned NADPH oxidase and thereby turning into a phenyl radical [47]. The radical itself harbors the potential to directly damage the DNA, with the subsequent activation of the DNA damage repair machinery. DPIHP, which is added to increase the degree of conversion, is usually accompanied by CQ, which has been shown to exert genotoxic potential by itself [49–51]. Even though the total mass of DPIHP, which is below 1%, does not significantly alter the cytotoxic properties of Transbond MIP, it most likely evokes γH2AX foci. A high genotoxic potential of diphenyliodonium chloride (DPIC), a substance closely related to DPIHP, has been previously reported based on the γH2AX assay [24]. Furthermore, the genotoxic activity of the substance itself and/or its degradation into halogenated benzenes has been mentioned [52]. Not surprisingly, the presence of the degradation product iodobenzene, as detected by gas chromatography [53], may be linked to the use of DPIHP. Nonetheless, DPIC and DPIHP share characteristics that seem to have a significant impact on their genotoxic properties. A putative synergistic effect of methacrylates, CQ and/or other additives in terms of ROS, and induction of DNA damage has been previously suggested [33].

Therefore, regarding the cytotoxic/genotoxic potential of a product, not only the manufacturer’s list of chemical composition must be taken into account but also the residues derived from the synthesis of the raw materials, which may affect the biocompatibility of the product in a negative manner. Therefore, all chemicals with a potential to exert endocrine activity, cytotoxicity, genotoxicity, and allergic reactions should be listed in the product safety profile [53–56]. In orthodontic treatment, a major exposition to monomers occurs during the application of brackets at the beginning of multiband therapy. The use of a rubber dam has long been advocated for orthodontic purposes since it can minimize the direct contact of unpolymerized adhesive with the buccal/oral mucosa and reduce systemic uptake [57]. Thorough air suction appears to be beneficial for patients as well as for the dental staff since the distribution, ingestion, and inhalation of free aerosol can be minimized. Since the long-term effects and accompanying safety issues remain unclear, the application of adhesives should be performed using the considered amounts, as excess material must be thoroughly removed and contact with the gingival, subgingival, and interproximal tissues must be avoided [11].

The use in dental practice of HEMA- and TEGDMA-free adhesives, e.g., silorane-based adhesives, which seem to be superior with respect to biocompatibility, has recently been suggested [39]. Regardless, a methacrylate-free product may still be dependent on common photoinitiating additives, such as CQ, DPIC, or DPIHP, which also need to be considered when assessing biosafety in terms of genotoxicity.

## Conclusions

Doubts regarding the biosafety of orthodontic adhesives have been raised due to the increasing number of reports addressing the leaching and cytotoxic and genotoxic properties of single ingredients. Therefore, it seemed reasonable to compare orthodontic adhesives in terms of their genotoxic and cytotoxic potential, both of which are closely related. Differences regarding the genotoxic potential were further elucidated with respect to single components conceivably responsible for the genotoxic effects observed. The DNA-damaging capability of adhesives was assessed with the notion that the co-initiator DPIHP likely has an additive effect on the γH2AX focus formation induced by methacrylate monomers like HEMA, TEGDMA, UDMA, and BisGMA. Therefore, DPIHP may enhance the genotoxicity of a compound. This compelling presumption will be subject of future investigations. The clinical relevance of the cytotoxic and genotoxic properties of orthodontic adhesives remains unclear due to the short exposure time and sharp decline in concentration resulting from dilution effects in the aqueous environment.
